# Time-resolved XUV ARPES with tunable 24–33 eV laser pulses at 30 meV resolution

**DOI:** 10.1038/s41467-019-11492-3

**Published:** 2019-08-06

**Authors:** Edbert J. Sie, Timm Rohwer, Changmin Lee, Nuh Gedik

**Affiliations:** Department of Physics, Massachusetts Institute of Technology, Cambridge, MA 02139 USA

**Keywords:** Characterization and analytical techniques, Superconducting properties and materials, Solid-state lasers

## Abstract

High harmonic generation of ultrafast laser pulses can be used to perform angle-resolved photoemission spectroscopy (ARPES) to map the electronic band structure of materials with femtosecond time resolution. However, currently it is difficult to reach high momenta with narrow energy resolution. Here, we combine a gas phase extreme ultraviolet (XUV) femtosecond light source, an XUV monochromator, and a time-of-flight electron analyzer to develop XUV-based time-resolved ARPES. Our technique can produce tunable photon energy between 24–33 eV with an unprecedented energy resolution of 30 meV and time resolution of 200 fs. This technique enables time-, energy- and momentum-resolved investigation of the nonequilibrium dynamics of electrons in materials with a full access to their first Brillouin zone. We evaluate the performance of this setup through exemplary measurements on various quantum materials, including WTe_2_, WSe_2_, TiSe_2_, and Bi_2_Sr_2_CaCu_2_O_8+δ_.

## Abstract (web summary)

Currently, it is difficult to reach high momenta with narrow energy resolution via laser-based angle-resolved photoemission spectroscopy (ARPES). Here, Sie et al. develop a time-resolved XUV based ARPES setup which can access the first Brillouin zone of all materials with narrow energy resolution.

## Introduction

Angle-resolved photoemission spectroscopy (ARPES) has the unique ability to resolve the electronic band structure of materials in momentum space. In this technique, photons of energy higher than the work function are used to eject the electrons from the surface of materials into the vacuum. By measuring the kinetic energies and momenta of these photo-ejected electrons, one can obtain the energy-momentum dispersion relation of the materials, including strongly correlated systems, topological insulators, and two-dimensional materials^1–4^. Typically, ARPES experiments are performed at synchrotrons, which can provide bright and tunable high-energy photons to map the equilibrium band structure. With the advent of femtosecond laser amplifiers and frequency harmonics through nonlinear crystals, ARPES can be performed using widely accessible, tabletop laser sources. As compared to the synchrotron-based ARPES, the laser light source provides better energy and momentum resolutions, yielding much sharper photoemission spectra^5,6^. The harmonics of Ti:Sapphire lasers at 6–7 eV is used as the light source^7,8^, which has also made it possible to take advantage of the femtosecond time resolution (10^−15^ s) in laser-based ARPES experiments. This is carried out using a pump-probe method to record an ultrafast movie of the electronic band structure after photo-excitation by another laser pulse^9,10^. Such time-resolved laser-based ARPES provides invaluable insights into the nonequilibrium electronic properties of matter^11,12^.

Although this technique has made the band structure mapping accessible in a time-resolved manner, it has several limitations. In particular, laser-based ARPES experiments through nonlinear crystals are limited to <7 eV photon energies. This puts a restriction on the range of accessible momentum values in reciprocal or k-space

1 $$\hbar k_\parallel = \sqrt {2{\it{m}}\left( {{\it{h\nu }} - \phi } \right)} \sin \theta$$

where *k*_||_ is the in-plane momentum of the electron, *m* is the free electron mass, *hv* is the photon energy, *ϕ* is the work function, and *θ* is the propagation angle with respect to normal incidence. For a large number of material systems, we are interested in the dynamics of electrons located near the edge of the Brillouin zone (BZ), which, however, is inaccessible using this method. Furthermore, the photoemission cross-section of element-specific orbitals can be very low at these energies for some material systems. For these reasons, there has been an increasing demand for time-resolved ARPES setups with photon energies higher than 7 eV^13–15^. The recently developed tabletop 10.5^16^ and 11 eV^17^ laser ARPES setups have helped to circumvent this issue. However, these techniques focused on achieving a higher photon-energy with high energy-resolution, and not on the femtosecond time-resolution. Moreover, the increasing interest in two-dimensional materials, such as the transition-metal dichalcogenides, necessitates light sources with photon energies larger than 18 eV to investigate electrons located at the corner of the BZ (~1.3 Å^−1^) by tilting the sample at a reasonable angle of 45°. In order to study electrons at deeper binding energies and wider momentum space, larger photon energies are required. Several groups have demonstrated the ability of XUV-based time-resolved ARPES^18–29^, particularly taking advantage of high-energy photons at a few-femtosecond resolution to investigate the ultrafast dynamics at BZ corners. However, the short time-resolution comes at a price of a rather broad energy-resolution in the order of a few hundred meV, and there have been significant efforts to achieve better energy resolution, ranging from 150 meV down to 60 meV^22–29^. In many cases, much narrower energy-resolution is required to disentangle subtle features in more complex band structures.

We develop a new technique that integrates an angle-resolved time-of-flight (ARTOF) electron analyzer and XUV femtosecond light source with photon energies that can be tuned selectively between 24, 27, 30, and 33 eV with a narrow energy resolution of 30 meV. This energy resolution marks a significant achievement for XUV-based time-resolved ARPES with previously reported resolution. This setup enables direct mapping of the entire band structure in most materials without the need to tilt the sample. In fact, by tilting the sample, this setup offers full access to the first BZ in all condensed matter systems.

## Results

### Instrument overview

**Fig. 1:**
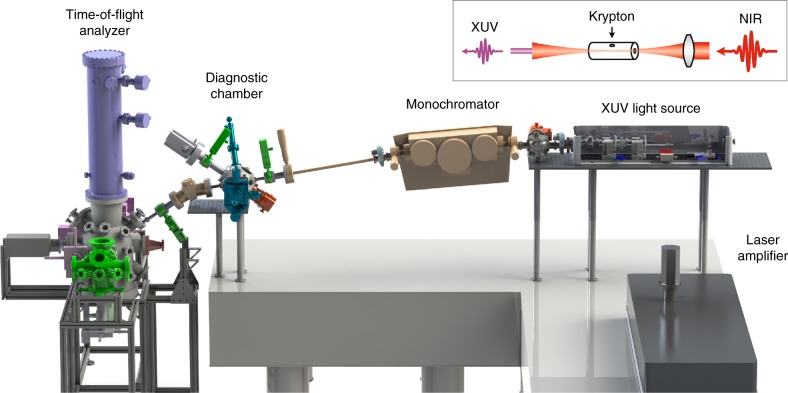
XUV-based time-resolved ARPES setup overview. Inset (top-right) shows the experimental layout for high harmonic generation (HHG) of a near infrared (NIR) light through a hollow fiber filled with krypton gas to generate extreme ultraviolet (XUV) light

Figure 1 shows the overview of our XUV time-resolved ARPES setup, comprising three major sections: (1) XUV light source, (2) XUV monochromator, and (3) XUV diagnostic chamber. At the XUV light source section, the laser amplifier output is used to drive a high harmonic generation (HHG) process and produce coherent XUV laser pulses. The monochromator is used to select one harmonic and further narrow the energy resolution using an adjustable exit slit. The diagnostic chamber is used to monitor the XUV photon energy, photon flux and spot size. Finally, we use a time-of-flight electron analyzer (Scienta ARTOF 10k)^30–32^, which provides three-dimensional dispersion (energy and two components of momentum) without the need to tilt the sample. The large, conical acceptance angle of the detector (up to ±18°) is particularly useful for obtaining both time-resolved and angle-resolved photoemission spectra with fast data acquisition. The resulting setup achieves a typical operating performance at photon energies of 24–33 eV, photon flux of 10^8^–10^9^ photons/sec (30 eV) at the sample at 30 kHz, time resolution of 200 fs, and energy resolution of 30 meV (33 eV). Below, we describe the roles of XUV light source, monochromator and diagnostic chamber in more detail.

### XUV light source

HHG is a nonlinear process in which high-order odd harmonics of the driving fundamental frequency pulses are coherently generated, typically in the XUV between 10–100 eV^33,34^. Detailed discussions on the physics of HHG process can be found in the references^35–39^. In our setup, the fundamental laser beam is focused into a hollow fiber that is filled with a nobel gas (Fig. 1, inset)^40^. Here we use a customized version of XUUS (KMLabs) with a much shorter fiber (1 cm) than typically used (5 cm) to optimize the lower-energy harmonics. We also made the XUUS assembly more compact to accommodate the diverging XUV output and maintain the time-preserving footprint on the grating. Finally, we added a cooling system surrounding the hollow-fiber assembly to improve the HHG stability with 7% RMS noise level. The ionization potential of the gas plays a crucial role in determining the cut-off photon energy and conversion efficiency of the XUV output. In particular, a noble gas with higher ionization potential increases the cut-off energy but reduces the conversion efficiency. Among the noble gases we experimented, we find that krypton has the best matching ionization energy to generate XUV harmonics at around 30 eV. In our setup, the HHG process is driven by a Ti:Sapphire laser amplifier (Wyvern 500, KMLabs) with photon energy of 1.59 eV (780 nm), pulse duration of 40 fs, and pulse energy of 300 µJ at 30 kHz repetition rate.

### XUV monochromator

ARPES energy resolution is determined largely by the photon energy bandwidth, apart from other significant factors such as the sample surface quality, energy analyzer settings, geometric alignment, and space-charge effects. Each harmonic produced by a 40-fs laser pulse at 780 nm in a hollow fiber has a typical linewidth in the order of 200 meV, which is still too broad to see detailed features in the band structure. Our monochromator is designed to achieve (i) narrow energy linewidth, (ii) short pulse duration, and (iii) high flux throughput. The minimum achievable values of the energy linewidth and pulse duration are related by the Fourier-transform limit of a light pulse. This can be expressed by the time-bandwidth product for a gaussian pulse

2 $${\mathrm{\Delta }}\tau \left( {{\mathrm{fs}}} \right){\mathrm{\Delta }}{\it{E}}\left( {{\mathrm{eV}}} \right) \ge 1.825\left( {{\mathrm{fs}} \thinspace {\mathrm{eV}}} \right)$$

where Δ*τ* is the pulse width and Δ*E* the energy linewidth. Finally, we must ensure sufficiently high photon flux at the sample for fast data acquisition, typically in the order of 10^8^ photons/sec or higher. The above three parameters serve as guidelines for designing our XUV monochromator.

**Fig. 2:**
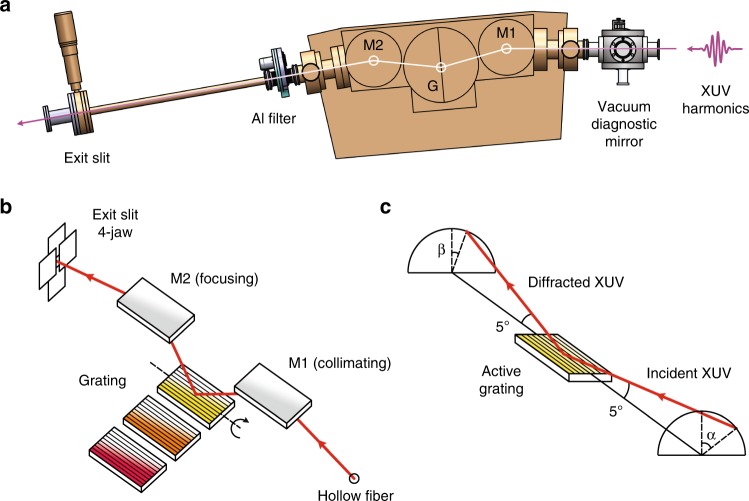
XUV monochromator. **a** Schematic of XUV monochromator in our setup. M1 and M2 refer to the collimating and focusing toroidal gold mirrors with respect to the grating G. **b** Optical layout inside the XUV monochromator. Three gratings with different groove density and blaze angle are placed inside the monochromator. A sliding mechanism inside the vacuum provides easy access to select the active grating, highlighted in yellow. **c** Light diffraction by an off-plane mounted (active) grating that forms cones with half-angle 5°

The principal design of our monochromator is inspired by the time-preserving single-grating concept of Luca Poletto’s group^41,42^, and implemented in collaboration with McPherson Inc with a spectral resolution of *R* = *λ*/Δλ = 1000 (see Methods). We implement the 5° grazing incidence angle and 800 mm focal length to achieve the optimum configuration between the flux throughput, energy resolution and time resolution. A sideview of our XUV monochromator (McPherson Inc) is shown in Fig. 2a. The grating is off-plane mounted (OPM) such that the groove lines are parallel to the plane of incidence^42^, and the resulting conical spectral dispersion forms an arc perpendicular to the plane of incidence (Fig. 2b, c). The OPM configuration at grazing incidence provides better flux throughput and less pulse width broadening compared to the conventional in-plane grating setup. The overall throughput of the system from source to sample—including three mirrors, a grating, and two filters—is about 10%.

### XUV diagnostic chamber

**Fig. 3:**
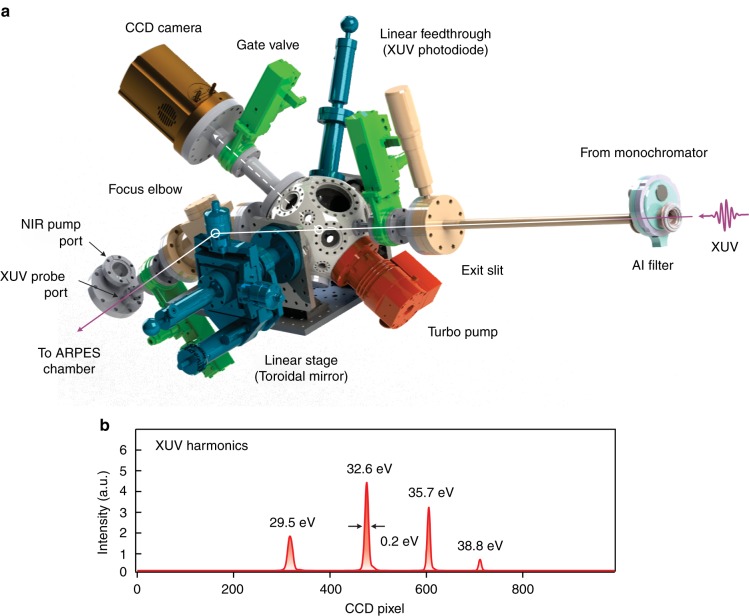
XUV diagnostic chamber. **a** Overview of the XUV diagnostic chamber in our setup. **b** XUV harmonics as imaged from the charge-coupled device (CCD) camera connected to the diagnostic chamber

We characterize the resulting XUV photon energy, photon flux, and spot size using a CCD camera and an XUV photodiode at the diagnostic chamber (Fig. 3a). This also allows us to monitor the HHG efficiency while optimizing the HHG fiber alignment (see Methods). The main spherical chamber (Kimball Physics) accommodates a toroidal mirror that can be inserted in and out of the beam path using a manual linear stage. When the toroidal mirror is inserted in the beam path, the XUV beam gets reflected and focused onto the CCD camera (ANDOR), displaying a series of XUV harmonics (Fig. 3b). When the toroidal mirror is removed from the beam path, we can insert the XUV photodiode to measure the photon flux. During ARPES measurements, the XUV beam passes through the chamber and enters the last section before the sample—the focus elbow. A toroidal mirror is mounted at the focus elbow, with a 1-to-1 imaging ratio, such that the diverging XUV beam from the exit slit is refocused onto the sample symmetrically. The resulting spot size at the sample is therefore adjustable by the exit slit.

### Evaluating instrument performance and material applications

We test the system performance through measurements of some transition-metal dichalcogenides (TMDs). This includes semimetal WTe_2_, semiconductor WSe_2_, and charge density wave material TiSe_2_. In addition, the measurements of a high-Tc superconductor Bi_2_Sr_2_CaCu_2_O_8+δ_ (Bi-2212) demonstrate the significance of the photon energy tunability of the setup. The obtained high-energy photons allow us to access the band structure in a wider range of the first BZ, and for some systems we are interested to investigate the electrons near the BZ corners.

### WTe_2_ semimetal

**Fig. 4:**
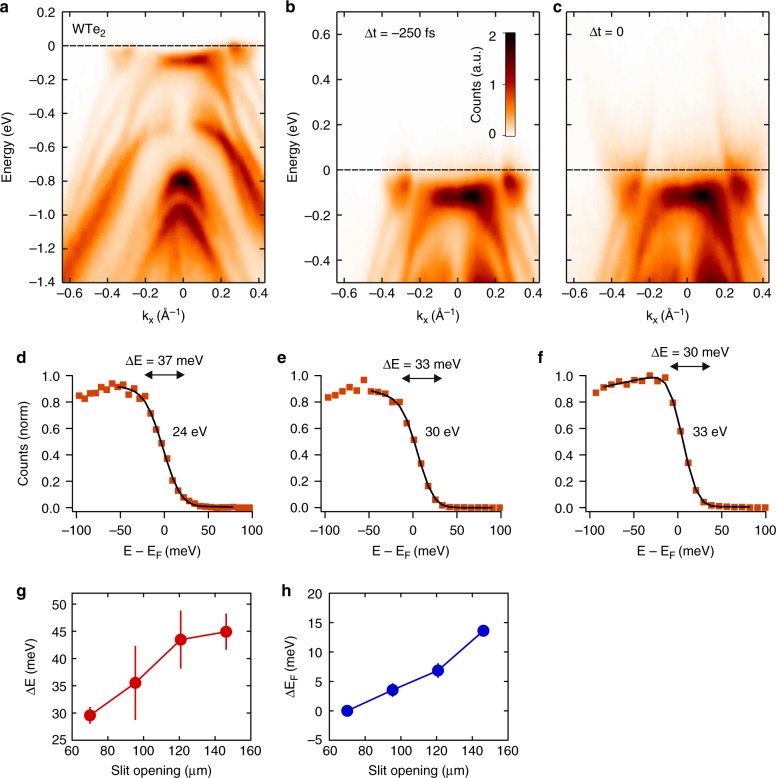
WTe_2_ semimetal. **a** ARPES spectra of WTe_2_ taken with 30 eV photons at 37 K. **b** Time-resolved ARPES results of WTe_2_ at 250 fs before the pump pulse arrives, and **c** at zero delay. **d**–**f** Energy resolution of the setup using 24, 30, and 33 eV photons. **g** Energy resolution (Δ*E*) of the WTe_2_ spectra taken with 33 eV photons at different slit openings. **h** The shift of the Fermi level (Δ*E*_*F*_) measured as function of slit opening. Error bars indicate the uncertainties of least square fitting of data to Eq. (3)

WTe_2_ is a layered semimetal TMD that crystalizes in a distorted hexagonal net with an orthorhombic unit cell (Td phase). Recently, this material is reported to exhibit a large non-saturating magnetoresistance^43^, a type-II Weyl semimetal^44^, and an ultrafast symmetry switch between the Weyl and Dirac phases^45^, garnering tremendous interest in the condensed matter physics community. The band structure of bulk WTe_2_ is calculated in reference^46^, which can be compared with our ARPES results. Figure 4a shows the band structure of WTe_2_ measured at 37 K using 30 eV probe pulses. We can identify the electron pocket at 0.3 Å^−1^ and hole pocket at 0.2 Å^−1^. In comparison, a 6-eV ARPES setup typically reaches a momentum space range of 0.19 Å^−1^. Figure 4b, c shows the time-resolved ARPES results before and after photoexcitation by a 1.59 eV pump pulse. The promoted electrons at higher-energy bands allow us to reveal the energy dispersion at energies beyond 500 meV above the Fermi level. In this way, the electron pockets are clearly revealed. By tuning the time delay between the pump and probe pulses, the carrier lifetimes can be measured and their dynamics can be studied for future investigation.

We can estimate the energy resolution of our XUV ARPES setup by plotting the energy distribution curve (EDC) of the electron pocket. The EDC is fitted to a Fermi–Dirac distribution convoluted with a Gaussian function

3 $${\hskip-1pt}{{I}}\left( {{E}} \right) = {{f}}\left( {E,T} \right){{D}}\left( E \right) \otimes g\left( {{{E}}{\prime} } \right) = \frac{{{{D}}\left( {{E}} \right)}}{{e^{\left( {E - E_F} \right)/{{k}}_{{B}}{{T}}} + 1}} \otimes e^{ - \left(4\ln 2\right){{E}}{\prime} ^{2}/{\mathrm{FWHM}}^2}$$

where *D*(*E*) is the density of states, *T* is sample temperature and FWHM is energy resolution. We use a 1200 grooves/mm grating for 24 eV measurements, and a 964 g/mm for 30 and 33 eV. We obtain an energy resolution of 30–37 meV after subtracting the thermal contribution in the Fermi–Dirac distribution (Fig. 4d–f). Here, we can tune the energy resolution between 30–45 meV by adjusting the exit slit opening between 70–146 µm (Fig. 4g). Correspondingly, the flux increases from 10^8^ to 10^9^ photons/sec. While the data clearly demonstrates that the slit improves the sharpness of ARPES spectra, it should be noted that the Fermi level shifts by ~15 meV by opening the slit to the same extent (Fig. 4h). The Fermi level shift likely arises from space-charge effects, in which the photoelectrons experience a Coulombic repulsion among themselves and result in a distorted ARPES spectrum. At increasing electron counts, the Fermi energy shifts upward, and the spectrum broadens. The space-charge effects in ARPES measurements have previously been observed not only with femtosecond pulses^47,48^, but also with picosecond pulses^49^ and at a synchrotron beamline^50^. These effects are especially pronounced in a low-repetition rate, short pulse measurement as a large number of photoelectrons are emitted within a short distance from one another. Therefore, the broader energy-resolution at wider slit opening values can be attributed to a combination of broader bandwidth of the HHG output and larger space-charge effects among the photoelectrons. On the other hand, the highest energy-resolution (30 meV) measurement is mainly limited by the HHG bandwidth and less from the space-charge broadening. This is because, under normal operation we photoemit less than 300 electrons/pulse from the sample within 0.1 mm spot size, corresponding to a space-charge broadening of about 5 meV^48^.

### Bi_2_Sr_2_CaCu_2_O_8+δ_ (Bi-2212) high-Tc superconductor

Bi-2212 (Tc = 96 K) is an extensively studied high-temperature superconductor^51^, which can be used to test the photon energy dependence of our setup. Since the early ARPES measurements of cuprates, ∼22 eV photons have been frequently used because this photon energy yields a sharp quasiparticle peak feature. The advantage of using the optimal photon energy is clearly indicated both from measurements^52^ and calculations^53^. The photon energy tunability of our setup also helps to mitigate the space-charge effects^47,48^. As we are mostly interested in the cuprate band structure near the Fermi level, tuning the photon energy to the most efficient harmonic order allows us to reduce the relative number of valence band photoelectrons that do not reach the time-of-flight detector, yet contribute to a significant amount of space-charge broadening during the photoemission process.

**Fig. 5:**
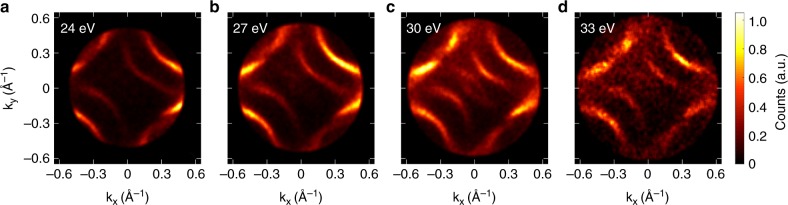
Bi-2212 high-Tc superconductor. Fermi surface contours of Bi-2212 measured using different photon energies at 30 K. Fermi energy contours were acquired by integrating over a 50-meV energy window with **a** 24, **b** 27, **c** 30, and **d** 33 eV photon energies. The 33-eV measurement was acquired in a relatively shorter time as compared to those of lower photon energies

Figure 5a–d shows the Fermi surface measurements at 30 K using four different photon energies (24, 27, 30, and 33 eV). At each photon energy, all four nodes of the Fermi arc are clearly visible, along with a partial view of the off-nodal regions. Shadow bands originating from the superstructure are also observed in all photon energies. Such features are especially prominent in 30-eV measurements (Fig. 5c). Although using 33-eV photons provides the largest range of momentum space, 24-eV is probably the most favorable choice due to the sharp quasiparticle intensity and weaker superstructure band contributions. This demonstrates the significance of photon energy tunability in the XUV regime provided by our setup. Such capability can be useful in resolving element-specific orbital bands in other materials with different photoemission cross-sections.

### WSe_2_ semiconductor

**Fig. 6:**
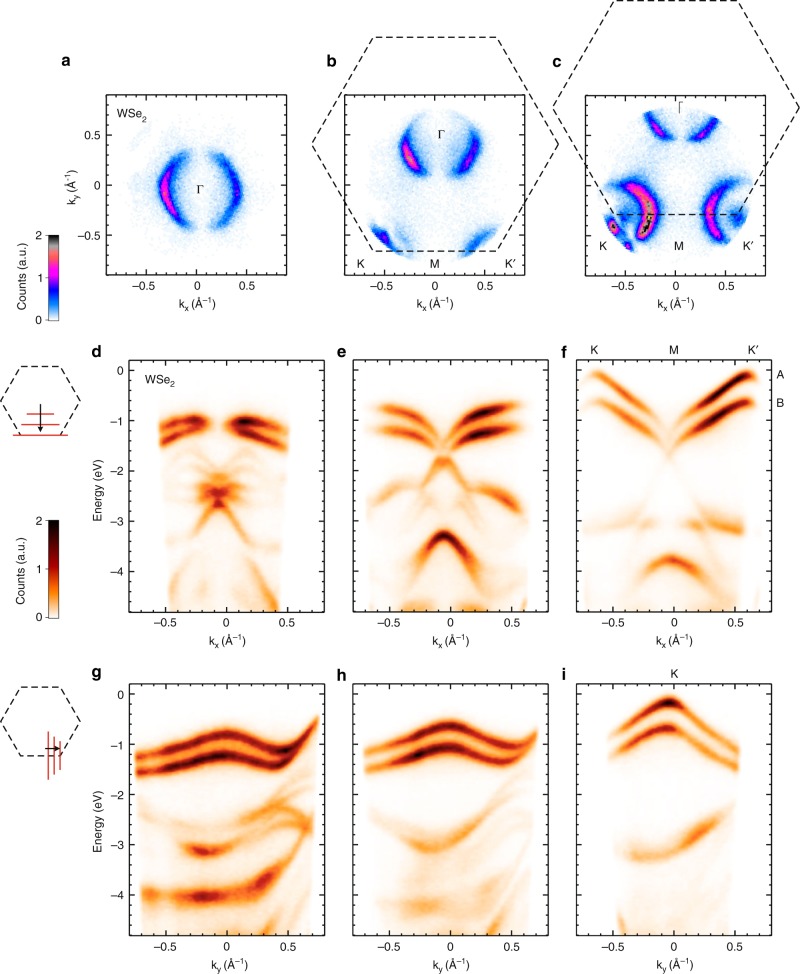
WSe_2_ semiconductor. ARPES measurement of bulk WSe_2_ with 30-eV photons at a sample temperature of 30 K. **a**–**c** constant energy surface at energy slightly below the valence band top. Subsequent panels from left to right are obtained by tilting the sample (0°, 9°, 18°) to reach the K and K′ valleys in the BZ. The right panel shows the A and B valence bands at the K and K′ valleys. **d**–**f** *E*(*k*_*x*_) linecuts as *k*_*y*_ is varied between Γ and M. **g**–**i** *E*(*k*_*y*_) linecuts as *k*_*x*_ is varied between M and K′

WSe_2_ is a layered semiconductor TMD that crystallizes in a centrosymmetric hexagonal structure (2H phase). The lowest conduction band in bulk WSe_2_ is located halfway along the Γ–K direction, denoted as Q valley (see below), and the highest valence band at Γ point, leading to an indirect gap with magnitude of 1.3 eV. Although much interest in the semiconducting TMDs is focused on their non-centrosymmetric monolayer counterparts, particularly on the unique spin-valley coupling and the large spin-split bands at the K and K′ valleys, the bulk counterparts also possess the essential elements of spin-valley coupling at the surface where inversion symmetry is broken. The WSe_2_ valleys are located at the zone corner of the BZ (1.3 Å^−1^)^54^, which can be used to demonstrate the large momentum range of our setup. Figure 6a–c shows the BZ map at energy slightly below the valence band maximum using 30-eV XUV pulses at 30 K. In this measurement, we tilt the sample progressively (at increasing tilt angles of 0°, 9°, 18° from left to right panels) in order to map the K and K′ valleys. The 30-eV XUV photons enable us to reach the zone corner, which cannot be achieved using 6-eV photons at any sample tilt. It also allows us to probe into deeper binding energies as we discuss below.

Figure 6d–i shows the energy dispersion along the (d–f) linecuts parallel to the K–M–K′ path in the BZ and the (g–i) linecuts perpendicular to it. This shows the advantage of using ARTOF detection to obtain 3D dispersion (*E*, *k*_*x*_, *k*_*y*_) without the need to continuously tilting the sample as compared to the conventional hemispherical analyzer. In this measurement, the time-of-flight analyzer is set at a high voltage configuration to access deeper binding energies beyond 4 eV. We can identify the two valence bands that are split by 540 meV due to strong spin-orbit coupling in this material. Inside the bulk, each of these valence bands corresponds to the up-spin and down-spin states altogether, originating from every two adjacent layers of WSe_2_. Contributions from the topmost WSe_2_ layer should mimic those of the monolayers, which could be disentangled using a surface-sensitive and spin-resolved ARPES technique^55^, as well as time-resolved photoemission^56^. The high energy-resolution of our setup enables a clear observation of avoided-crossing fine structures as can be seen below the Fermi level.

**Fig. 7:**
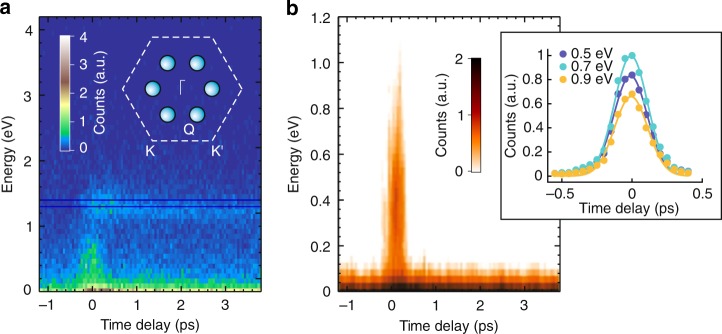
Time-resolved ARPES results in WSe_2_. **a** Population lifetime of the excited electrons in the Q valleys. Inset: Brillouin zone of WSe_2_ showing the six inequivalent Q valleys located in between Γ and K points. **b** Photon-dressed states emerging within the bandgap, taken at higher energy-resolution and narrower energy window. Inset: Photoemission counts as a function of time delay, taken at three different energy cuts

Photoexcitation can promote electrons from the valence bands to occupy the higher-lying conduction bands using 1.59 eV pump pulses, whose energy is larger than both direct (K→K) and indirect (Γ→Q) gaps in bulk WSe_2_. Due to the low symmetry of this point, there are 6 inequivalent Q valleys in the BZ. By using time-resolved ARPES with 1.59 eV pump and 30 eV probe we can study the dynamics of photoexcited electrons, covering the entire possible excitations within ±1.59 eV with respect to the highest occupied levels. In particular, the spectra can access momenta >0.5 Å^−1^ at a binding energy of 1.59 eV (Fig. 6d–i). Figure 7a shows that the photoexcited electrons are found to quickly accumulate at the Q valleys at energy 1.3 eV above the highest valence band and stay there with lifetime longer than 3 ps. In addition, there is a very short-lived signal at time-zero at energy within the gap. Figure 7b shows a higher energy-resolution measurement taken within the gap. We attribute this signal to a possible photon-dressed replica of the lower valence bands that are induced by the pump pulse, which opens a new avenue for future investigation. A closer look into the faster component at different energies reveal that the data can be fitted well with a single Gaussian function. The colored dots and lines in the inset of Fig. 7b represent time-resolved photoemission data, and the corresponding Gaussian fits, respectively. The least square fits to the data lead to an experimental time-resolution of 198 ± 14 fs.

### TiSe_2_ charge density wave material

TiSe_2_ is a layered TMD with 1T lattice structure. This material is considered to be a correlated system that possesses rich features. It exhibits a charge density wave (CDW) second-order phase transition at *T*_*c*_ ~ 200 K into a commensurate 2 × 2 × 2 superstructure. The driving force for the CDW transition has been ascribed to either a Jahn–Teller effect, an excitonic-insulator transition, an antiferromagnetic transition or a mixture of them, as the resulting energy gains from these mechanisms are well justified. A comprehensive review discussing the CDW transition of this compound and related TMD materials can be found in reference^57^. While the origin of the CDW transition has been much studied in equilibrium, the experiments in references^21,58^ have shown a new perspective on the CDW formation under non-equilibrium conditions. By using ultrashort laser pulses in an ARPES experiment, the long-range order that gives rise to the CDW band folding is found to exhibit an ultrafast breakdown within 20 fs. This timescale is much faster than the lattice response expected in this material and ascribed to photoinduced screening by the excited carriers. This result is consistent with the CDW mechanism that is purely excitonic driven.

**Fig. 8:**
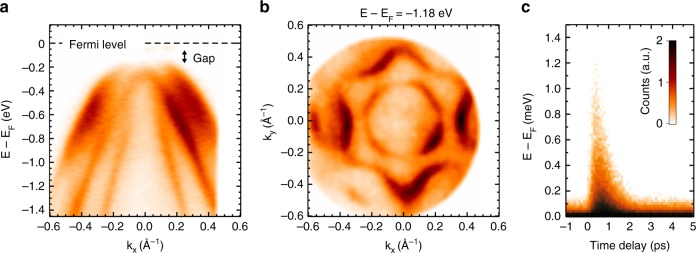
TiSe_2_ charge density wave system. **a** TiSe_2_ spectra showing the separation of the Se-4p_x_, 4p_y_, 4p_z_ orbitals. **b** Constant energy surface at 1.18 eV below the Fermi energy demonstrating clear hexagonal structure. **c** Momentum-integrated excitation transient; the rise time is resolution limited at 200 fs. The measurements were performed at a sample temperature of 30 K

These early experiments demonstrated the potential of time-resolved ARPES to probe the microscopic mechanisms by studying the nonequilibrium phase transitions. However, their energy resolution of 400 meV is too broad to monitor the change in CDW gap upon photoexcitation. The XUV monochromator presented here does, in principle, have the necessary energy resolution to achieve this, which opens a new avenue for future investigation. We show a preliminary result on the TiSe_2_ band structure measured at 30 K using 30-eV ARPES in Fig. 8a–c. The ARPES data in Fig. 8a show distinct bands originating from Se-4p_x_, 4p_y_, and 4p_z_ orbitals, as well as the 100-meV bandgap at the Γ-point due to the backfolding with the Ti-3d band from the M-point^21^. These bands disperse differently along different momentum directions, which form a circle and hexagonal warping in the constant energy surface at 1.18 eV below the Fermi energy (Fig. 8b). By applying 1.59 eV pump pulse and scanning the pump-probe time delay, we can monitor the carrier relaxation dynamics at energy above and below the Fermi energy. The carrier population time-trace at various energy levels is shown in Fig. 8c in a momentum-integrated manner. The fast rise time of 200 fs is consistent with the instrument time-resolution, while the slow decay shows that the excited carriers relax with lifetime on the 2 ps timescale.

### Operation mode

The acquisition time for a single ARPES map (3D band structure as functions of *E, k*_*x*_, *k*_*y*_) is 66 min for all the static data shown in Figs. 4–8 (WTe_2_, WSe_2_, Bi-2212, TiSe_2_). For the time-resolved ARPES data, the acquisition times are 7 h 30 min for WSe_2_ (Fig. 7a, b, 100 steps at 50-fs interval) and 4 h 30 min for TiSe_2_ (Fig. 8c, 60 steps at 100-fs interval). The ARTOF detection is limited to 1 electron/pulse during data acquisition to avoid multiple near-simultaneous hits of the X, Y readout at the delay-line detector. This limits the maximum tolerable XUV flux of our instrument. For example, by considering the typically used 2.6% fractional space angle (±13° cone) and assuming 0.01–0.1 electron/photon yield at 30 eV^59,60^, we get a maximum tolerable XUV flux of 10^7^–10^8^ photons/sec at 30 kHz. In order to overcome this limit, we can choose to repel incoming photoelectrons energy-selectively by using a bias voltage in front of the detector, e.g. 1–2 eV energy window admittance. This increases the maximum tolerable XUV flux to about 10^8^–10^9^ photons/sec at 30-meV resolution for all the data we have shown. The pump spot size is 200 µm with fluences of 0.6 mJ/cm^2^ (WTe_2_, Fig. 4c), 1.2 mJ/cm^2^ (WSe_2_), 1.6 mJ/cm^2^ (TiSe_2_).

## Discussion

Finally, we discuss technical constraints that limit the energy and time resolutions of our setup. The energy resolution can in principle be improved by (i) using a higher groove density grating, (ii) increasing monochromator focal length, and (iii) narrowing the exit slit. However, manufacturing higher groove density gratings with sub-micrometer to nanometer scale pitch can be very costly, and usually suffer from lower light reflectivity and spectral impurities, such as ghost effects, satellites, and diffuse scattering^61^. A longer monochromator focal length leads to a wider light footprint on the toroidal mirrors and grating. This requires fabricating a set of larger XUV optics inside the monochromator and the overall length scale of the setup, which can be a challenging contest to meet the budget and lab space constraints. A narrow exit slit may improve the energy resolution up to the spectral resolution limit of the monochromator determined by the grating groove density and the focal length. This is at the expense of reducing the photon flux at the output. Meanwhile, the HHG process in our setup is driven using a laser amplifier at 300 µJ pulse energy, which is much smaller than the pulse energy used in typical HHG setups reported elsewhere (1–10 mJ). Hence, the 30-meV energy resolution of our setup is obtained after optimizing the above parameters. Meanwhile, the time resolution of our setup is limited by the diffracted XUV optical delay at the grating (~130 fs), the non-collinear pump-probe geometry (~60 fs), and the NIR pump pulse broadening by the dispersive optical elements. This results in an overall time resolution close to 200 fs (see Methods).

In conclusions, we develop an XUV-based time-resolved ARPES setup using tunable 24–33 eV photon energies with 30-meV energy resolution and 200-fs time resolution. We show exemplary photoemission spectra from three TMD samples (WTe_2_, WSe_2_, and TiSe_2_) and a high-Tc superconductor Bi-2212, providing full access to their first BZ. Photoexcitation with 1.59 eV pump pulses demonstrates the time-resolved capabilities of the setup to enable high-resolution band structure mapping at energies above the Fermi level, carrier relaxation dynamics studies in the energy-momentum space, and electronic phase transition investigation in TMDs.

## Methods

### XUV monochromator

The principal design of our monochromator is inspired by the time-preserving single-grating concept of Luca Poletto’s group^42^, and implemented in collaboration with McPherson Inc. A sideview of our XUV monochromator manufactured by McPherson is shown in Fig. 2a. The main chamber of the monochromator comprises two toroidal mirrors (M1, M2) and a stack of three gratings (G1, G2, G3), all optics are gold-coated and mounted at 5° grazing angle configuration. We implement the X-ray Czerny-Turner (XCT) design that has a symmetric optical layout with respect to the grating. There are two arms—one between the light source and M1, the other one between M2 and the exit slit—each has a nominal focal length of 800 mm and dimension of 85 × 30 × 12 mm. M1 and M2 are identical but 180° rotated with respect to each other. Their toroidal surfaces are curved such that the XUV light is collimated toward the grating. We select three gratings with different blaze angles and groove densities, to operate the monochromator at three different photon energies and resolutions: 500 g/mm (blaze angle 4.7°), 964 g/mm (14.6°), and 1200 g/mm (26.7°). Each grating has a dimension of 75 × 30 × 9.1 mm. The three gratings are mounted in parallel on a sliding stage for quick switching inside the vacuum. In this experiment, the grating is mounted such that the groove lines are parallel to the plane of incidence. This is called an OPM^42^ grating configuration, and the resulting conical spectral dispersion forms an arc perpendicular to the plane of incidence (Fig. 2b, c). The OPM configuration at grazing incidence provides better flux throughput (grating efficiency of about 25%) and less pulse width broadening compared to the conventional in-plane grating setup. The active grating is mounted on a motorized rotation stage with the rotation axis coinciding with the surface of the grating oriented along the plane of incidence. The energy linewidth of the outgoing beam can be reduced by narrowing the exit slit horizontally. The dispersions at the slit using different gratings are 2.5 nm/mm (500 g/mm), 1.3 nm/mm (964 g/mm), 1.0 nm/mm (1200 g/mm). Separate from this design, various monochromator configurations can be considered for future improvements in the time-resolution, energy-resolution, and throughput^62–64^.

The reflectivity of XUV light is very sensitive to the incidence angle, photon energy and polarization, which can be theoretically estimated through calculations using the Fresnel equations^65,66^. Here, the incidence angle is measured relative to the surface and not to the surface normal. For example, at 45° incidence angle, 30 eV photon energy and light polarization parallel to the surface, the estimated reflectivity of a gold-coated mirror is 22%; meanwhile, at 5° grazing incidence angle, the reflectivity is 85%. The incidence angle and focal length are crucial parameters that determines the overall performance of the monochromator. We first define the desired spectral resolution of *R* = *λ*/Δλ = 1000 and the photon flux of 1 × 10^10^ photons/sec at the exit slit. As we use lower incidence angle and longer focal length, the mirror reflectivity and the dispersion increase respectively. However, the beam footprint could increase beyond the optics and result in a significant photon loss. Moreover, as the transverse footprint increases, more grating lines are illuminated and the diffraction lines are sharper. On the other hand, the diffracted optical delay between the footprint edges can contribute to the pulse width broadening. For this reason, we implement the 5° grazing incidence angle and 800 mm focal length to achieve the optimum configuration between the flux throughput, energy resolution and time resolution. The XUV has a divergence of ~1 mrad at 30 eV from the fiber output, with footprint of 12 × 1 mm at zero-order on the grating and the mirrors. Our optics have a larger dimension to allow a larger divergence (or footprint) for longer wavelength upgrade. The overall throughput of the system from source to sample—including three mirrors, a grating, and two filters—is about 10%.

Finally, there is a drastic pressure difference between the XUV light source (~10^2^ torr) and the ARPES measurement chamber (<10^−10^ torr). All optical components of the XUV beamline are housed in a vacuum chamber, including the XUV monochromator. In order to maintain the ultra-high-vacuum (UHV) condition at the ARPES chamber, we installed two 100-nm thick aluminum filters (by Lebow Company) that suppress a major flow of the noble gas, as well as three turbo pumps in consecutive stages of differential pumping—one right after the XUUS light source, another at the XUV monochromator, and the other at the XUV diagnostic chamber.

### Measuring XUV photon flux

The XUV photons are absorbed at the photodiode and create electron-hole pairs that will register into an electric current from which we can determine the photon flux. For instance, the conversion efficiency of the photodiode is 0.26 A/W at photon energy of 30 eV. Hence, a measured current of 12 nA corresponds to a photon flux of 1 × 10^10^ photons/sec per harmonic. This flux is measured after propagating through a fully opened exit slit from the monochromator. Comparable flux is obtained for other photon energies in the 24–33 eV range when optimizing to the particular harmonic^67^.

### Time resolution

The time-resolution of our setup is determined by the 780 nm pump pulse and the XUV probe pulse. Note that the output from our laser amplifier is immediately split using a thin beam-splitter such that 95% of the reflected beam is used to produce the XUV probe pulse and 5% of the transmitted beam propagates through the pump arm. At the pump arm, we introduce a pair of lenses to maintain the propagating beam diameter, a waveplate and a polarizer to adjust the pump fluence, a lens to focus the pump beam at the sample, and an optical viewport at the ARPES chamber. These dispersive optical elements introduce pulse width broadening of the pump beam. Meanwhile, the XUV pulse width broadening is dominated by the number of grating lines *N* illuminated by the XUV beam. In other words, a larger transverse footprint at the grating leads to a larger pulse width broadening. Finally, the 780 nm pump beam and the XUV probe beam intersect at an angle of 10.85° between each other. The normal vector of an untilted sample is aligned with the ARTOF detector line at an angle of 56° with respect to the XUV probe beam, and 45.15° with respect to the 780 nm pump beam. We can calculate the contributions of these factors to time resolution as follows. The Fourier-transform limited pulse width of a 30-meV bandwidth pulse is 60 fs, given by Eq. (2). If we use grating 964 g/mm, an effective transverse footprint of 1 mm, and operating at 30 eV (41.33 nm), the 1st-order diffracted optical path delay is *λN* ~ 40 µm. Hence, diffracted optical delay contribution from the grating amounts to ~130 fs. Meanwhile, the non-collinear time smearing contribution at the sample is 100 µm × sin(10.85°)/c ~ 60 fs, where the XUV spot size at the sample is 100 µm. Hence, the time-resolution of our setup is limited by the diffracted optical delay at the grating and the non-collinear pump-probe geometry, resulting in an overall time resolution close to 200 fs.

## Supplementary information

Source Data

## Data Availability

The authors declare that all data supporting the findings of this study are available within the paper.
